# Studying the Spatial Distribution of Physiological Effects on BOLD Signals Using Ultrafast fMRI

**DOI:** 10.3389/fnhum.2014.00196

**Published:** 2014-04-01

**Authors:** Yunjie Tong, Blaise deB. Frederick

**Affiliations:** ^1^McLean Imaging Center, McLean Hospital, Belmont, MA, USA; ^2^Department of Psychiatry, Harvard University Medical School, Boston, MA, USA

**Keywords:** BOLD fMRI, multiband EPI, physiological noise, independent component analysis, low frequency oscillations

## Abstract

The blood-oxygen-level dependent (BOLD) signal in functional MRI (fMRI) reflects both neuronal activations and global physiological fluctuations. These physiological fluctuations can be attributed to physiological low frequency oscillations (pLFOs), respiration, and cardiac pulsation. With typical TR values, i.e., 2 s or longer, the high frequency physiological signals (i.e., from respiration and cardiac pulsation) are aliased into the low frequency band, making it hard to study the individual effect of these physiological processes on BOLD. Recently developed multiband EPI sequences, which offer full brain coverage with extremely short TR values (400 ms or less) allow these physiological signals to be spectrally separated. In this study, we applied multiband resting state scans on nine healthy participants with TR = 0.4 s. The spatial distribution of each physiological process on BOLD fMRI was explored using their spectral features and independent component analysis (ICA). We found that the spatial distributions of different physiological processes are distinct. First, cardiac pulsation affects mostly the base of the brain, where high density of arteries exists. Second, respiration affects prefrontal and occipital areas, suggesting the motion associated with breathing might contribute to the noise. Finally, and most importantly, we found that the effects of pLFOs dominated many prominent ICA components, which suggests that, contrary to the popular belief that aliased cardiac and respiration signals are the main physiological noise source in BOLD fMRI, pLFOs may be the most influential physiological signals. Understanding and measuring these pLFOs are important for denoising and accurately modeling BOLD signals.

## Introduction

In blood-oxygen-level dependent (BOLD) functional MRI (fMRI), the BOLD signal reflects temporal fluctuations in the blood, including blood volume, blood oxygenation, and blood flow. All of these factors are affected by regional neuronal activation, which increases local metabolic rate and oxygen consumption, and thereby leads to detectable BOLD signal changes. However, other physiological processes also induce changes in BOLD and are sometimes the dominant component in the BOLD signals (Murphy et al., 2011). The main physiological processes seen in BOLD data are physiological low frequency oscillations (pLFOs; In this manuscript, we use pLFOs to represent the physiological part of LFOs, nLFOs to represent neuronal part of LFOs, and LFOs to represent the combination of both), respiration, and cardiac pulsation. These different physiological processes confound the BOLD signals in different ways. Understanding the mechanisms and spatial distributions of their impacts on BOLD is critical in: (1) revealing the real neuronal signal; (2) better understanding and modeling the BOLD signal itself; and (3) extracting useful physiological information from fMRI.

Among these processes, the most mysterious and understudied are the pLFOs, which are intrinsic spontaneous oscillations of roughly 0.1 Hz. Despite many hypotheses, their origins and functions are not fully understood (Wise et al., 2004; Julien, 2006; Shmueli et al., 2007; Birn et al., 2008; Chang et al., 2009; Aalkjaer et al., 2011; Murphy et al., 2011). We have recorded the time courses of pLFOs in the periphery (e.g., fingertip and toes), and found that they resemble concurrent BOLD signals in the brain with certain time delays (Tong et al., 2012). This implies that the pLFOs are systemic signals that travel with the blood. Because the origin of these signals is unclear, there is no standard method to measure the pLFOs. Moreover, the spectrum of pLFOs overlaps that of the intrinsic neuronal signals (~0.1 Hz) in BOLD fMRI (i.e., nLFOs). These factors complicate the study of how pLFOs impact the BOLD fMRI. Although respiration (~0.3 Hz) and cardiac pulsation (~1 Hz) (Tortora and Derrickson, 2008) signals have clear origins and distinct spectral signatures, it is also difficult to directly assess the effects of these processes in the BOLD signal because the typical sampling period of fMRI is longer than 2 s. This only allows us to fully sample fluctuations with a frequency of 0.25 Hz (or 0.167 Hz in the case of a 3-s TR) and lower. Any signal with higher frequency content will be aliased into the BOLD signal, which is the case for cardiac pulsation (~1 Hz) and the majority of the respiration signal (0.2 – 0.4 Hz). Despite some efforts to understand the effects of these processes (Glover et al., 2000; Desjardins et al., 2001; Beall and Lowe, 2007; Birn et al., 2008; Chang et al., 2009; Frederick et al., 2012), these aliased physiological signals are hard to separate out and study individually. Furthermore, they are mixed up with the pLFOs, which make it even harder to study pLFOs’ effects.

Recently, a new fMRI acquisition sequence has been developed that dramatically decreases the repetition time of fMRI to hundreds of milliseconds (Feinberg et al., 2010; Moeller et al., 2010; Setsompop et al., 2012; Xu et al., 2012) in full brain coverage. As a result, the two main physiological signals (i.e., respiration and cardiac pulsations) can be fully sampled. This allows us to explore the impact of these physiological signals on the BOLD fMRI individually. In this study, we spectrally separated the BOLD fMRI from a resting state acquisition into three bands: LFOs, respiration, and cardiac pulsation. We then mapped the effects of each process on BOLD fMRI with two approaches: (1) using their distinct spectral signatures to separate their impacts on BOLD spatially, and (2) applying independent component analysis (ICA) (Beckmann and Smith, 2004; Beckmann et al., 2005), a data driven method that is capable of separating the various “significant components” of the BOLD signal automatically (i.e., “neuronal” and “physiological” components in LFOs then can be identified by visual inspection) (Tohka et al., 2008; Kelly et al., 2010). In this manuscript, we are more interested in the physiological components calculated by ICA.

## Materials and Methods

### Protocols

Functional MRI resting state studies were conducted in nine healthy participants (five males, four females, average age ± SD, 34 ± 12.7 years). In the resting state studies, participants were asked to lie quietly in the scanner and view a gray screen with a fixation point in the center. The resting state scans lasted 360 s. The Institutional Review Board at McLean Hospital approved the protocol, and participants were compensated for their participation.

All MR data was acquired on a Siemens TIM Trio 3 T scanner (Siemens Medical Systems, Malvern, PA, USA) using a 32-channel phased array head matrix coil. After acquiring a high resolution localizer image (ME-MPRAGE, TR/TI/TE = 2530/1100/3.31, 256 × 256 × 128 voxels over a 256 mm × 256 mm × 170 mm sagittal slab, GRAPPA factor of 2), multiband EPI (University of Minnesota sequence cmrr_mbep2d_bold R008) (Feinberg et al., 2010) data were obtained with the following parameters: TR/TE = 400/30 ms, flip angle 43°, matrix = 64 × 64 on a 220 mm × 220 mm FOV, multiband factor = 6, 30 3.0 mm slices with 0.5 mm gap parallel to the AC–PC line extending down from the top of the brain.

For each participant, the standard FSL fMRI pre-processing steps, including motion correction, high pass filter (>0.01 Hz), slice time correction, and spatial smoothing (5 mm) were applied to the original BOLD data [using FEAT v6.00 of FSL 5.04 (Jenkinson et al., 2012)] prior to further analysis.

### Data analysis

#### Spectral method

**Figure 1:**
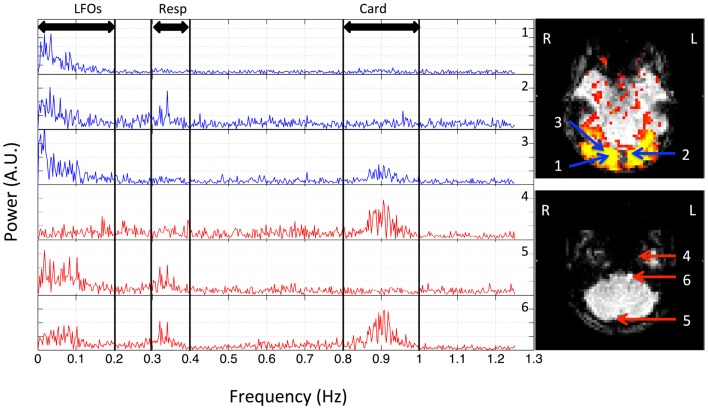
**Power spectra of BOLD signals from six voxels, which were selected from the resting state data (TR = 0.4 s) of one participant, and their locations in the brain**. Three distinct spectral ranges corresponding to different physiological processes were marked. The two graphs on the right indicate the locations of these voxels in the visual cortex (top) and bottom slices (lower).

In order to understand the spectral signatures of different physiological signals, the power spectrum of BOLD signal at each voxel was calculated for each participant, and these subject-specific spectral signatures were defined based on the participant’s own power spectra. Figure 1 shows the spectra of six typical BOLD signals from one participant. Three main signals were found (for this participant) and marked as: (1) LFOs (<0.2 Hz); (2) respiration (0.3–0.4 Hz); (3) cardiac pulsation (0.8–1.0 Hz). For the spatial distribution of these signals in the brain, we first defined a baseline in the spectra (i.e., the averaged power obtained from the range where the stable and minimum power was observed). For example, in Figure 1, the baseline was the averaged value of the spectrum ranging from 0.4 to 0.7 Hz. If the peak power in the frequency band of a certain process (i.e., LFOs, respiration, cardiac pulsation) exceeded twice the baseline level, the corresponding voxel was considered as the voxel influenced by this process. Sometimes, voxels can be affected by one, two, or three processes. The spatial map of certain process (for each participant) is obtained by combining all the corresponding voxels affected by this process. The averaged result maps of nine participants were all projected onto the standard brain.

#### ICA method

**Figure 2:**
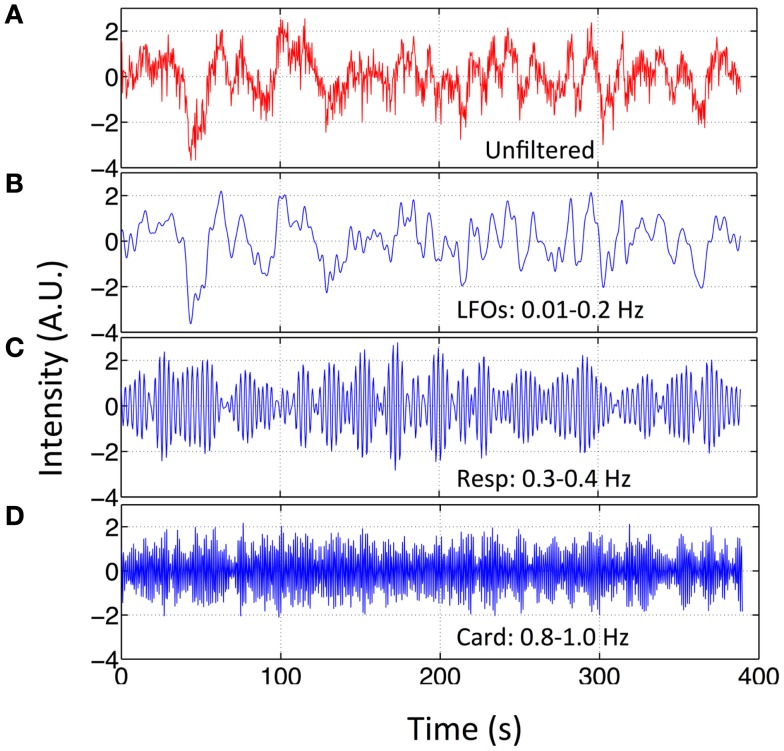
**Original BOLD fMRI (TR = 0.4 s) data and its filtered versions**. Temporal trace of the original BOLD data (in red) from resting state fMRI scan (TR = 0.4 s) in **(A)** and its band-passed versions in **(B)** 0.01–0.2 Hz; **(C)** 0.3–0.4 Hz; and **(D)** 0.8–1.0 Hz.

Independent component analysis was used to assess the impact of different physiological signals on BOLD fMRI. First, we spectrally separated resting state data sets of these nine participants. For each participant, in addition to the original data set (unfiltered), three more filtered data sets were generated from the original multiband data using the zero delay Fourier domain filter (MATLAB, The Mathworks, Natick, MA, USA). The filter’s ranges are: (1) 0.01–0.2 Hz for LFOs; (2) roughly 0.2–0.4 Hz for respiration, the band-pass filter range was chosen on subject-specific respiration frequency that varies from participant to participant (e.g., for participant in Figure 1, it is 0.3–0.4 Hz); (3) roughly 0.8–1.2 Hz for cardiac signals, same as respiration signal, the band-pass filter range was chosen on subject-specific cardiac frequency (e.g., for participant in Figure 1, it is 0.8–1.0 Hz). The subject-specific respiration and cardiac pulsation ranges for all the participants can be found in Figure S1 in Supplementary Material. As result, each filtered data set corresponds to only one main process. This is possible due to the high sampling rate of the multiband EPI sequence (TR = 0.4 s). We chose the upper limit of LFOs to be 0.2 Hz for the two reasons; first, to maximize the low frequency band, based on previous research (Niazy et al., 2011), which showed that the spectral range of LFOs in BOLD is beyond the 0.1-Hz and can be as high as 0.2 Hz (Boyacioglu et al., 2013); and secondly to exclude two other physiological signals, namely respiration and cardiac pulsation. For most of the participants, 0.2 Hz is below the respiration frequency (definitely below cardiac frequency). Figure 2 shows the original temporal traces of one BOLD signal (TR = 0.4 s) in red and its band-pass filtered versions in blue (same participant as in Figure 1), which corresponded to LFOs, respiration, and cardiac pulsation. We then used group multivariate exploratory linear optimized decomposition into independent components (MELODIC 3.10) ICA from FSL on the original and spectrally separated resting state data sets (four data sets, each has nine participants) independently. The dimensionality of MELODIC was set to 35, a typical number of components for ICA studies (Beckmann and Smith, 2004). Within the 35 ICs found in the unfiltered data set, seven commonly recognized ICs with physiological (non-neuronal) origins were identified. They are classified into four categories based on their main locations and characteristics of the patterns: (1) ventricular system; (2) vascular system; (3) white matter, and (4) motion artifact. Then, we searched the corresponding physiological ICs from the results of LFOs, respiration, and cardiac pulsation. Different dimensionalities were also used to test the generalization of the resulting spatial patterns. By associating each data set with certain physiological patterns, we hope to understand location and mechanism, by which, different physiological process affect the BOLD signals.

## Results

### Spectral signatures of different physiological processes

The spectra of several typical voxels from a participant’s resting state scan are plotted in Figure 1. Due to the high temporal resolution of the multiband sequence (TR = 0.4 s), the upper limit of the spectrum is 1.25 Hz. The top three graphs (in blue) are from the voxels located in the visual cortex (the region of visual cortex was localized by a separate block-design checkerboard stimulus on the same participant in the same imaging session). The top graph on the right indicates the locations of these voxels (blue arrows) in the visual cortex (marked by yellow patterns). The last three graphs are from the voxels from the bottom slices (axial) of the same data set, which, based on its extracerebral location, are likely to present the physiological fluctuations from incoming arteries, exiting veins, or mixture of both (voxels size: 3.5 mm × 3.5 mm × 3.5 mm). The bottom graph on the right indicates the locations of these voxels (red arrows) in the bottom slices. They were selected from the areas overlapping with the main vasculatures. The power of the signals mainly resides in three spectral regions for all the participants: (1) the low frequency region; (2) the respiration region; and (3) the cardiac region. This is clearly demonstrated in Figure 1 for one participant, where these three spectral regions are <0.1, 0.3–0.4, and 0.8–1.0 Hz, respectively. However, these components are not equally represented in all the voxels, indicating different physiological processes influence the voxels differently. For example, for the voxels from the lower voxels around the incoming arteries, that are more prone to physiological fluctuations due to their locations, there are voxels with three distinct spectral components (voxel 6), with only the respiration and low frequency components (voxel 5), and with only cardiac component (voxel 4). Similar effects were observed for the voxels from the visual cortex. Even though they all have the low frequency component, cardiac and respiration signals were also sometimes observable, to varying degrees. Lastly, among these signals, LFOs and cardiac signals generally have more signal power than that of respiration. These findings in Figure 1 are widely presented in all the participants with subject-specific frequency ranges. The spectra of representative voxels (all from the bottom slices) for the rest of the participants were shown as Figure S1 in Supplementary Material. In addition to showing the subject-specific frequency ranges of these processes (especially for respiration and cardiac pulsations), they also demonstrated that: (1) these processes are not equally represented in all the voxels, as we discussed (for instance, there are no respiration signals observed in the representative voxels from participant three and eight); (2) LFOs and cardiac signals generally have more power than that of respiration.

### Spatial distributions of different physiological processes (spectral analysis)

**Figure 3:**
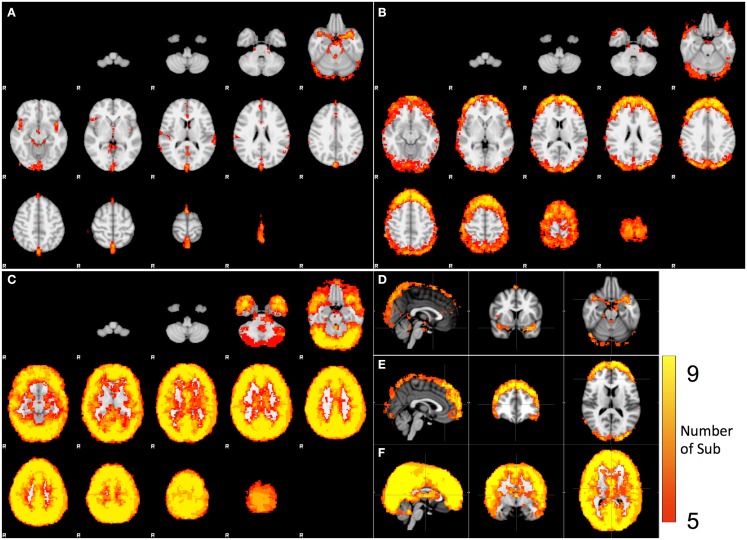
**Spatial distribution of the voxels that have significant spectral content in the following bands: (A) cardiac pulsation, (B) respiration, and (C) LFO**. The orthogonal views of **(A,B,C)** are shown in **(D,E,F)**.

The spatial distributions of cardiac, respiration, and LFOs signals calculated from nine participants are plotted in Figures 3A–C. For each distribution map, a color was assigned to each voxel, indicating the number of participants from which the physiological process was observed in this voxel. Figures 3D–F are the same maps displayed orthogonally. The spatial distributions of these three physiological signals are different. Figures 3A,D indicate the brain regions that have significant contribution from cardiac pulsation. These regions are closely associated with cerebral vasculature, as demonstrated previously (Dagli et al., 1999; Beall and Lowe, 2007), including the areas with high density of arteries at the base of the brain (e.g., Circle of Willis, middle cerebral artery), and the areas with big veins (superior sagittal sinus). This is because that cardiac signal is carried by the blood vessels (as a pressure wave), especially by the arteries. Similar pulsation patterns were identified by Boubela et al. (2013) using temporal ICA on the ultrafast resting state fMRI. Respiration affected mainly the prefrontal and occipital lobes, as well as the top of the brain, as shown in Figures 3B,E. Most voxels in the brain are affected by the LFOs. In the spectral study, we could not separate the pLFOs from nLFOs. However, ICA would allow us to separate these effects based on their characteristic spatial distributions, which are independent from other signals. The detail was discussed in next section.

### Spatial distributions of different physiological processes (ICA)

**Figure 4:**
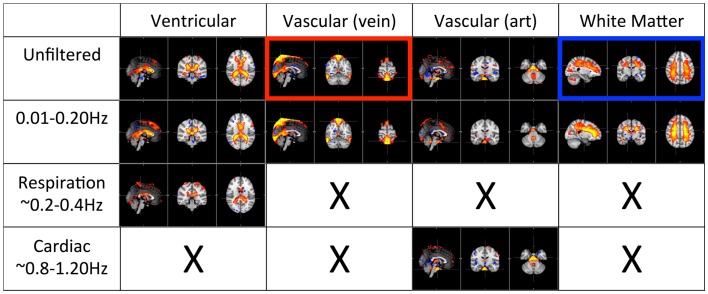
**Four prominent physiological ICs identified from the unfiltered data (in top panel) and their corresponding ICs selected from the ICA result of the filtered data**. The patterns associated with cerebral vascular and white matter are marked with red and blue boxed respectively.

**Figure 5:**
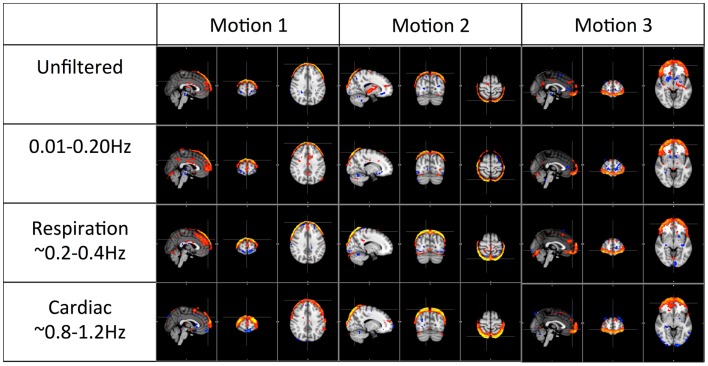
**Three prominent motion-related physiological ICs identified from the unfiltered data (in top panel) and their corresponding ICs selected from the ICA result of the filtered data**.

Figures 4 and 5 shows the most prominent physiological patterns identified from unfiltered data (in top panels). They included the patterns resembling main ventricles, main cerebral vasculature (both veins and arteries), white matter (as in Figure 4), and motion artifacts (as in Figure 5). The corresponding ICs from the filtered data sets (i.e., pLFOs, respiration, and cardiac pulsation) were identified and listed accordingly. From Figure 4, we can see that not all the physiological processes produced the same physiological ICs. For instance, the cerebral vascular pattern (marked with the red box in Figure 4), can not be found in the ICA results of respiration and cardiac pulsation; the same behavior was observed for the white matter pattern (marked with the blue box in Figure 4).

Figure 5 shows three typical motion artifacts selected by ICA from each data set. On the contrary of Figure 4, similar patterns were produced by ICA from each data set.

## Discussion

### Effects of respiration assessed by spectral features

Respiration signals are distributed in the anterior, posterior, and superior regions of the brain, primarily at the cortical surface, and have no clear associations with the cerebral vasculature (Figures 3B,E). The salient feature of the respiration distribution is the circular and semi-circular pattern, which can be seen in the axial images in Figures 3B,E. In these images, the patterns comprising full circles can be observed at the top of the head. Gradually they become semi-circles, surrounding the prefrontal and occipital lobes. These patterns are consistent with these signals being the combination of several motion artifacts. For example, the circular pattern observed at the superior slices is likely to be caused by translational motion along the vertical axis (*Z*-axis in scanner coordinates), while the semi-circular pattern at the surface of the frontal and occipital cortex might be caused by rotation around the horizontal axis (*Y* -axis in scanner coordinates), i.e., a nodding motion. These motions are breathing-related, as respiration tends to move the head in these dimensions, a fact that is clearly observed in the motion traces generated during pre-processing. The data used here were motion-corrected by pre-processing in FSL (Jenkinson et al., 2002), however, the simple rigid body motion correction (six dimensions) is not enough to fully remove the effects of motion caused by breathing (for example, spin history effects). Therefore, using motion parameters generated in the pre-processing as noise regressors in the fMRI analyses is strongly suggested to covary out residual effects in the data. The respiration process likely affects the BOLD signals in other ways, such as by changing the oxygenation of the blood directly. For example, Birn et al. (2008) has demonstrated that the respiration depth affected BOLD fMRI signal.

### Effects of physiological processes assessed by ICA

**Figure 6:**
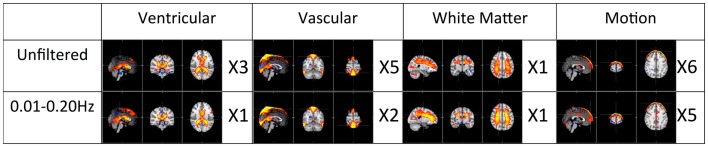
**Total number of physiological ICs identified from unfiltered vs. filtered data (0.01–0.2 Hz) is categorized and listed accordingly**.

The first panel of Figure 4 shows four prominent physiological ICs from unfiltered data. They are clearly associated with the ventricular system, cerebral vascular system (veins), cerebral vascular system (arteries), and white matter. These ICs are widely observed in ICA studies. Because ICs are derived from individual frequency bands, it is now possible to link them with different physiological processes. First of all, even after filtering out the physiological signals of respiration and cardiac pulsation completely, we are still able to detect every physiological IC (as shown in the second panel from the top in Figure 4) as seen with the unfiltered data (top panel). This demonstrates that LFOs are not 100% neuronal signals; on the contrary, they are likely associated with more than one physiological process that affects many regions of the brain (i.e., cerebral vasculature, ventricles, and white matter). Furthermore, in the ICs calculated from respiration and cardiac pulsation, certain spatial patterns associated with physiological ICs, including the cerebral vasculature (veins) and white matter patterns described above, were not observed. This continued to be the case even after we lowered the dimensionality in the ICA to prevent the splitting effects. This finding shows that pLFOs in BOLD are mostly – perhaps even solely – associated with the spatial patterns that result from the cerebral blood circulation. The present finding is consistent with our previous studies on BOLD pLFOs. For example, we found that the BOLD pLFOs, together with their temporal shifts, can be used to track cerebral blood flow in the brain (Tong and Frederick, 2010). Figure 4 also shows that respiration contributed to some brain regions, such as ventricles, while cardiac pulsation contributed to signal fluctuations in the arteries, as expected. However, the ICs from pLFOs matched the result of unfiltered data the best, confirming that pLFOs alone contribute significantly to some physiological noise components in the BOLD. If this is the case, the immediate question is what we gain by filtering out the respiration and cardiac signals (which is possible when using the multiband sequence). To explore this, we visually inspected and categorized all the ICs from unfiltered and filtered (LFOs) data. The result is shown in Figure 6, in which we can see the total number of physiological ICs identified from these two data sets. For instance, three ICs associated with the ventricular system were identified in the ICA results of the unfiltered data, whereas only one was identified from the result of the filtered data. There are five ICs associated with cerebral vasculature from the unfiltered data compared to two from the filtered data. In summary, out of 35 ICs, 15 were identified as physiological ICs from unfiltered data and nine from the filtered data. This indicates that by removing respiration and cardiac pulsation signals (even with a simple band-pass filter), less physiological-related ICs were produced, thus possibly increasing the sensitivity toward detecting the more subtle neuronal-based resting state networks (RSNs). Recently, there have been studies on spectral characteristics of the RSNs using ultrafast fMRI in which the spectral range of the RSNs were found to be much wider than is commonly believed (Niazy et al., 2011; Boubela et al., 2013; Boyacioglu et al., 2013). Moreover, ultrafast fMRI allowed people to use temporal ICA to identify physiological ICs and RSNs (Smith et al., 2012; Boubela et al., 2013).

Figure 5 shows the three prominent motion-related ICs selected from unfiltered data (in the top panel) and their corresponding ICs identified from the data of pLFOs, respiration, and cardiac pulsation. In contrast to what was shown in Figure 4, these motion ICs defined by circular patterns can be found independently in every data set. One unlikely possibility is that these different physiological processes caused the same motion. As we know from Figure 3, respiration causes visible global motion, whereas cardiac pulsation and pLFOs do not (they may cause regional motions). The other explanation is that any motion (regardless of its causes) recorded by BOLD would affect the entire spectrum, i.e., the spectrum of impulsive motions (spikes) is broad. This is why, from these three spectra, the same motion artifact ICs were detected individually. To validate this hypothesis, we applied ICA on a data set with yet another spectral band (0.4–0.8 Hz). The same motion artifacts, as shown in Figure 5, were found in the new ICA result.

## Conclusion

Fast fMRI acquisition greatly expands the spectral range of the BOLD fMRI (up to 1.25 Hz in this study). It has allowed us to explore the impacts of physiological processes on the BOLD signal. We have been able to map the spatial distributions of these different physiological processes, demonstrating that the cardiac pulsation is seen most prominently in the base of the brain where arteries are concentrated, while respiration affects prefrontal and occipital lobes through breathing-related motion. Most importantly, we found that the effects of pLFOs dominated many physiological patterns found in ICA, which suggests that, contrary to the popular belief that aliased cardiac and respiration signals are the main physiological noise in BOLD fMRI, pLFOs may be the most influential physiological signals. Understanding and characterizing these pLFOs are important in denoising and accurately modeling BOLD signals.

## Conflict of Interest Statement

The authors declare that the research was conducted in the absence of any commercial or financial relationships that could be construed as a potential conflict of interest.

## Supplementary Material

The Supplementary Material for this article can be found online at http://www.frontiersin.org/Journal/10.3389/fnhum.2014.00196/abstract

Figure S1 **Power spectra of representative BOLD signals from the rest eight subjects**. These voxels are all from the bottom slices of each subject. Three frequency bands corresponding to LFOs (in black), respiration (blue), and cardiac pulsation (red) are marked in each spectrum.
