# Supplementary figures and images for: Studying the Spatial Distribution of Physiological Effects on BOLD Signals Using Ultrafast fMRI
Source: Front Hum Neurosci. 2014 Apr 1;8:196. doi: 10.3389/fnhum.2014.00196 (PMC3978361; doi:10.3389/fnhum.2014.00196)

LFOs;

Respiration;

Cardiac pulsation;

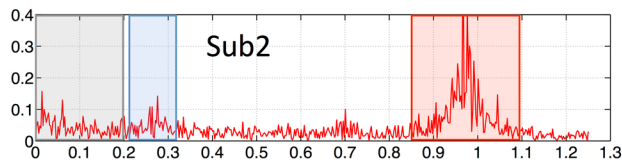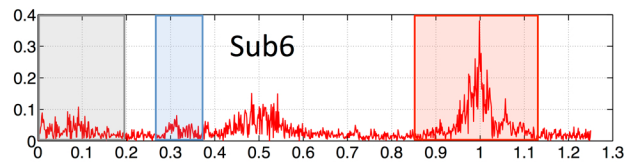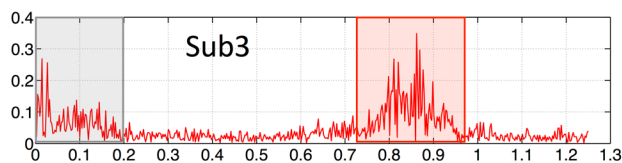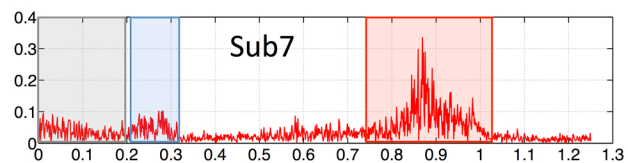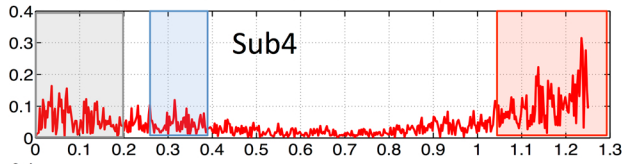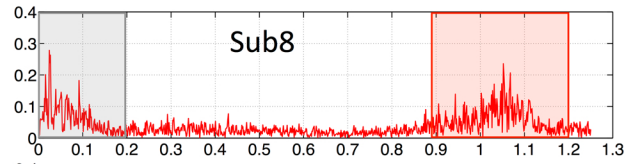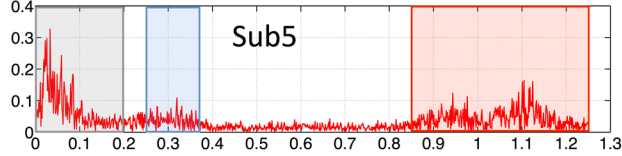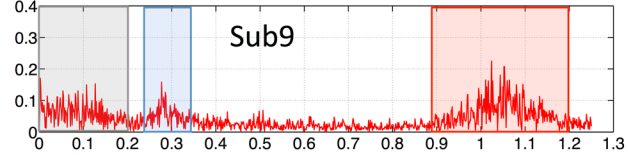

Frequency (Hz)

Supplement: Figure S1 — Power spectra of representative BOLD signals from the rest eight subjects. These voxels are all from the bottom slices of each subject. Three frequency bands corresponding to LFOs (in black), respiration (blue), and cardiac pulsation (red) are marked in each spectrum. [file Presentation_1.PDF]
